# Elevated expression of CXCL3 in colon cancer promotes malignant behaviors of tumor cells in an ERK-dependent manner

**DOI:** 10.1186/s12885-023-11655-y

**Published:** 2023-11-29

**Authors:** Yao Cheng, Xinyan Yang, Lichun Liang, Hua Xin, Xinyu Dong, Weidong Li, Jie Li, Xiaoli Guo, Yue Li, Jian He, Chunbin Zhang, Weiqun Wang

**Affiliations:** 1 Basic Medical College, Jiamusi University, Jiamusi 154002, Heilongjiang, China; 2 Clinical Laboratory, Beidahuang Industry Group General Hospital, Harbin 150088, Heilongjiang, China; 3 First Affiliated Hospital, Jiamusi University, Jiamusi 154002, Heilongjiang, China; 4 Department of Medical Technology, Collaborative Innovation Center for Translation Medical Testing and Application Technology Zhangzhou, Zhang Zhou Health Vocational College, Zhangzhou 363000, Fujian Province, China

**Keywords:** colon Cancer, CXCL3, MAPK/ERK, Malignant behavior

## Abstract

**Background:**

CXC chemokine ligand 3 (CXCL3) is a member of CXC-type chemokine family that is identified as a major regulator in immune and inflammation responses. Recently, numerous evidence indicated that CXCL3 is broadly expressed in various human tumor types, and it is also known to play a critical role in mediating tumor development and progression. However, the expression profile of CXCL3 and the exact molecular mechanism behind the role of CXCL3 in colon adenocarcinoma (COAD) has not been fully elucidated.

**Methods:**

The expression and clinical significance of CXCL3 mRNA and protein in the tissues from COAD patients were estimated using bioinformatics and immunohistochemistry assays. The expression and roles of exogenous administration or overexpression of CXCL3 in HT-29 and SW480 COAD cells were determined using enzyme-linked immunosorbent assay(ELISA), Cell Counting Kit-8 (CCK-8) and Transwell assays. Mechanically, CXCL3-induced malignant behaviors were elucidated using western blotting assay and extracellular signal-regulated protein kinase 1/2 (ERk1/2) inhibitor PD98059.

**Results:**

The cancer genome atlas **(**TCGA)-COAD data analysis revealed that CXCL3 mRNA is highly expressed and has high clinical diagnostic accuracy in COAD. Increased expression of CXCL3 mRNA was associated with patient’s clinical stage, race, gender, age, histological subtype, nodal mestastasis and tumor protein 53 (TP53) mutation status. Similarly, immunohistochemistry assay also exhibited that CXCL3 protein in COAD tissues was significantly up-regulated. Gene expression associated assay implied that CXC chemokine ligand 1 (CXCL1) and CXC chemokine ligand 2 (CXCL2) were markedly correlated with CXCL3 in COAD. Protein-protein interaction (PPI) analysis revealed that cyclin B1 (CCNB1), mitotic arrest deficient 2 like 1 (MAD2L1), H2A family member Z (H2AFZ) and CXCL2 may be the important protein molecules involved in CXCL3-related tumor biology. Gene set enrichment analysis (GSEA) analysis revealed that CXCL3 was mainly enriched in the cell cycle, DNA replication, NOD-like receptors, NOTCH and transforming growth factor-β (TGF-β) Signal pathways. In vitro, exogenous administration or overexpression of CXCL3 resulted in increased malignant behaviors of HT-29 and SW480 cells, and down-regulation of CXCL3 expression inhibited the malignant behaviors of these tumor cells. In addition, overexpression of CXCL3 affected the expression of genes related to extracellular signal regulated kinase (ERK) pathway, including ERK1/2, p-ERK, B-cell lymphoma-2 (Bcl-2), Bcl-2-associated X protein (Bax) and Cyclin D1. Finally, CXCL3-induced malignant behaviors in HT-29 and SW480 cells were obviously attenuated following treatment with ERK inhibitor PD98059.

**Conclusion:**

CXCL3 is upregulated in COAD and plays a crucial role in the control of malignant behaviors of tumor cells, which indicated its involvement in the pathogenesis of COAD.

**Supplementary Information:**

The online version contains supplementary material available at 10.1186/s12885-023-11655-y.

## Background

Colon adenocarcinoma (COAD) is one of the leading causes of cancer deaths worldwide, which is the second most common prominent cancer among women and the third in men, respectively [1]. It was estimated that more than one million individuals develop COAD worldwide each year and the disease-related mortality for COAD corresponds to about 33% [2]. In developing countries, around one quarter of patients with COAD are in an advanced stage at diagnosis, and thus have lost their opportunities for obtaining a radical surgery [3]. Even though the consensus policy in the diagnosis and treatment of COAD has developed rapidly in recent years, it is still an important issue because of its high prevalence and poor prognosis [4]. Therefore, novel prognostic biomarkers and therapeutic targets for COAD are urgently needed in order to overcome these problems.

Chemokines, also defined as chemotactic cytokines, are a class of secretory proteins with a low-molecular weight of 8 to 12 KD, which are initially chartered to play pivotal roles in inducing chemotaxis of various kinds of cells [5]. Chemokines contain a total of over 50 species, which can be classified into four subfamilies, CXC, CC, C and CX3C, depending on the location of cysteine residues [6]. It has been suggested that chemokines can restrain anti-tumor immunity by enabling infiltration of endothelial cells or their precursors towards the tumor microenvironment (TME), where these cells participate in the formation of new blood vessels that may provide oxygen and nutrient supply for rapidly growing tumors [7–9]. In additon, chemokines have also been demonstrated to facilitate anti-tumor immunity through their ability to recruit and activate multiple types of immune cells in the TME [7–9].

CXCL3 belongs to the CXC family of chemokines and can be further known as a ELR + CXC chemokine due to the presence of a conserved Glu-Leu-Arg sequence. CXCL3 exerts its biological functions mainly related to its interaction with CXC-chemokine receptor 2 (CXCR2) [7]. Our previous studies illustrated that CXCL3 was up-regulated in tissues with cervical cancer and prostate cancer, and exogenous administration or overexpression of CXCL3 dramatically stimulated tumor cell malignant behaviors via the mitogen-activated protein kinase (MAPK)/ERK and phosphoinositide 3-kinase (PI3K)/ protein kinase B (Akt) pathways [10–12]. Consistently, it has been recognized that CXCL3 and its receptor CXCR2 were highly expressed in hepatocellular carcinoma, esophageal cancer, and invasive breast cancer [13–15], and CXCL3/CXCR2-mediated signaling was crucial for the variation in the expression of numerous tumor-related genes, which is an essential event that is extremely implicated in tumor progression [16]. The purpose of this study is to determine the clinical significance, the biological role and underlying mechanism of CXCL3 in COAD, it is possible to provide a guideline for the clinical diagnosis and treatment of COAD.

## Materials and methods

### Bioinformatics analysis

The original CXCL3 mRNA-seq data including 284 COAD and 41 normal colon tissues were obtained from the Cancer Genome Atlas (TCGA) database (https://portal.gdc.cancer.gov/), and the expression levels of CXCL3 mRNA in tissues were analyzed using the university of alabama at birmingham cancer data analysis portal (UALCAN) database (http://ualcan.path.uab.edu/). Receiver operating characteristic (ROC) curve was used to calculate the area under the curve (AUC). The genes closely related to CXCL3 in COAD were obtained from UALCAN database, and the top 25 genes positively correlated with CXCL3 were displayed in the form of heatmap. The protein interaction network (PPI) was constructed through String database (https://cn.string-db.org/) and Cytoscape Software, and the tumor immune estimation resource (TIMER) database (https://cistrome.shinyapps.io/timer/) was used to determine the correlation between CXCL3 expression and CXCL3-related genes expression (or immunocyte infiltration). The TCGA-COAD data set was used for GSEA analysis, both p value and false discovery rate (FDR) q value less than 0.05 was considered to be significantly rich.

### Immunohistochemistry

Immunohistochemistry was conducted to detect the protein expression of CXCL3 in a commercial human colon tissue microarray (bioaitech). Briefly, the microarray was incubated with an antibody targeted to CXCL3 (1:100; ImmunoWay) at 4˚C overnight, followed by treatment with a SABC Kit (Boster) according to the supplier’s instructions. After color development with 3,3′-diaminobenzidine (DAB), Hematoxylin was applied for nuclear counterstain. CXCL3 staining was scored using the sum of the following two indicators: (1) the intensity of staining: negative-weak (1), medium (2), strong (3) and super strong (4); (2) the percentage of staining cells: ≤25% (1), > 25–≤50% (2), > 50–≤75% (3) and > 75% (4). The final score = (1)+(2). Low expression was defined as a final score ≤ 4 and high expression with a final score ≥ 5.

### Cell lines and cultures

Human CC HT-29 and SW480 cells were provided by ATCC. Cells were grown in RPMI-1640 medium (for HT-29) or L-15 medium (for SW480) containing 10% fetal bovine serum (FBS; Biological Industyies) and 100 U/ml penicillin/streptomycin, and the medium containing FBS with penicillin/streptomycin was referred to as complete medium.

### Cell transfection

Following construction of empty lentiviral vector and lentiviral expression vector harboring CXCL3 gene sequence or interfering sequence targeted CXCL3, the pseudovirus particles containing these vectors were produced by GeneCopoeia Inc. (Rockville, MD). CXCL3-overexpressing cells, CXCL3-deficiency cells and their mock control cells were established by infection of the pseudovirus particles according to the supplier’s recommendations.

### ELISA analysis

A total of 4 × 10^5^ cells were cultured in each well of six-well plates containing 1 ml serum-free medium. After 24 h of inoculation, the supernatant was obtained by centrifuging the medium. The concentration of CXCL3 in supernatant was measured and calculated using an ELISA Kit (Mskbio) in strict accordance with the manufacturer’s protocol. The experiments were repeated in triplicate.

### Cell proliferation analysis

Cell counting kit-8 (CCK-8) reagent (Med Chem Express) was employed for cell proliferation experiments: (a) Exogenous experiment: 4 × 10^3^ HT-29 or SW480 cells were seeded in each well of a 96-well plate containing different concentrations of recombinant CXCL3 (peprotech). (b) Endogenous experiment: 4 × 10^3^ CXCL3-overexpressing or CXCL3-deficiency HT-29 or SW480 cells, as well as their mock control cells were seeded in each well of a 96-well plate containing 100 µl of complete medium. In addition, the complete medium supplemented with DMSO (control) or the ERK1/2 blocker PD98059 was utilized to assess whether the role of CXCL3 is mediated by ERK1/2 pathway. After 48-hour culture in the experiments, 10 µl of CCK8 reagent was added to each well, and the OD value of each well was measured after 40 min of reaction. The experiments were repeated in triplicate.

### Cell migration analysis

Cell migration analys1s was performed using 24-well Transwell systems (Biofil). (a) Exogenous experiment: 3 × 10^4^ HT-29 or SW480 cells in serum-free medium were seeded in each upper chamber of Transwell, while the bottom chamber was filled with 20% FBS medium containing different concentrations of recombinant CXCL3. (b) Endogenous experiment: 3 × 10^4^ CXCL3-overexpressing or CXCL3-deficiency HT-29 or SW480 cells, as well as their mock control cells in serum-free medium were seeded in each upper chamber of Transwell, while the bottom chamber was filled with 20% FBS medium. Similarly, 20% FBS medium supplemented with dimethyl sulfoxide (DMSO) (control) or the ERK1/2 blocker PD98059 was also utilized in the endogenous experiment. After 48-hour culture in the experiments, the migratory cells that had invaded through the filter were fixed and stained with crystal violet solution in ethanol. The experiments were repeated in triplicate.

### Cell clone analysis

2 × 10^2^ cells were grown in each well of a 24-well plate containing 500 µl of complete medium. After 2 weeks of routine culture, the medium was discarded, and the plate was soaked twice with PBS followed by staining with 0.1% crystal violet for 40 min. The number of colonies was counted under an eletron microscope. The experiments were repeated in triplicate.

### Western blot analysis

A total of 30 µg protein extracts was resolved by 12% sodium dodecyl sulfate-polyacrylamide gel electrophoresis (SDS-PAGE) and subsequently electroblotted onto polyvinylidene difluoride (PVDF) membranes. Following blocking with 5% skimmed milk, the membranes were treated with various specific antibodies, including a rabbit antibody against ERK1/2 (1:1,000 dilution; Abways Technology Company), a rabbit antibody against p-ERK1/2 (1:1,000 dilution; Abways Technology Company), a rabbit antibody against Bcl-2 (1:1,000 dilution; Abways Technology Company), a rabbit antibody against Bax (1:1,000; Abways Technology Company), a rabbit antibody against Cyclin D1 (1:1,000; Abways Technology Company) and a mouse antibody against β-actin (1:1,000; Zhongshan Golden Bridge Bio-technology, Beijing). The relative intensity of ERK1/2, Bcl-2, Bax and Cyclin D1 was normalized to β-actin, whereas p-ERK1/2 was normalized to total ERK1/2. Immunoreactive protein bands were detected using 5200 multifunctional imaging system (Tanon Science & Technology Co., Ltd., Shanghai, China), and the intensity of each band was quantified using image analysis software (version 4.2; Tanon Science & Technology Co., Ltd., Shanghai, China). The experiments were repeated in triplicate.

### Statistical analysis

Statistical analyses were executed with SPSS statistics 22.0 software. Mann-Whitney U test was used for comparing differences in the expression of CXCL3 mRNA-seq data. Univariate and multivariate Cox regression models were used to analyze the prognostic significance of CXCL3 in patients with COAD. The correlation between two-group was analyzed with Pearson correlation analysis, and comparison of the rates was conducted using the Pearson Chi-square test. Comparisons between the two-group in the cell experiment were made using T test or Rank sum test, whereas multiple-group comparisons were assessed by one-way ANOVA and LSD test. Differences with *P* values < 0.05 were considered significant.

## Results

### CXCL3 mRNA is up-regulated in COAD tissues and has high accuracy in clinical diagnosis

**Fig. 1:**
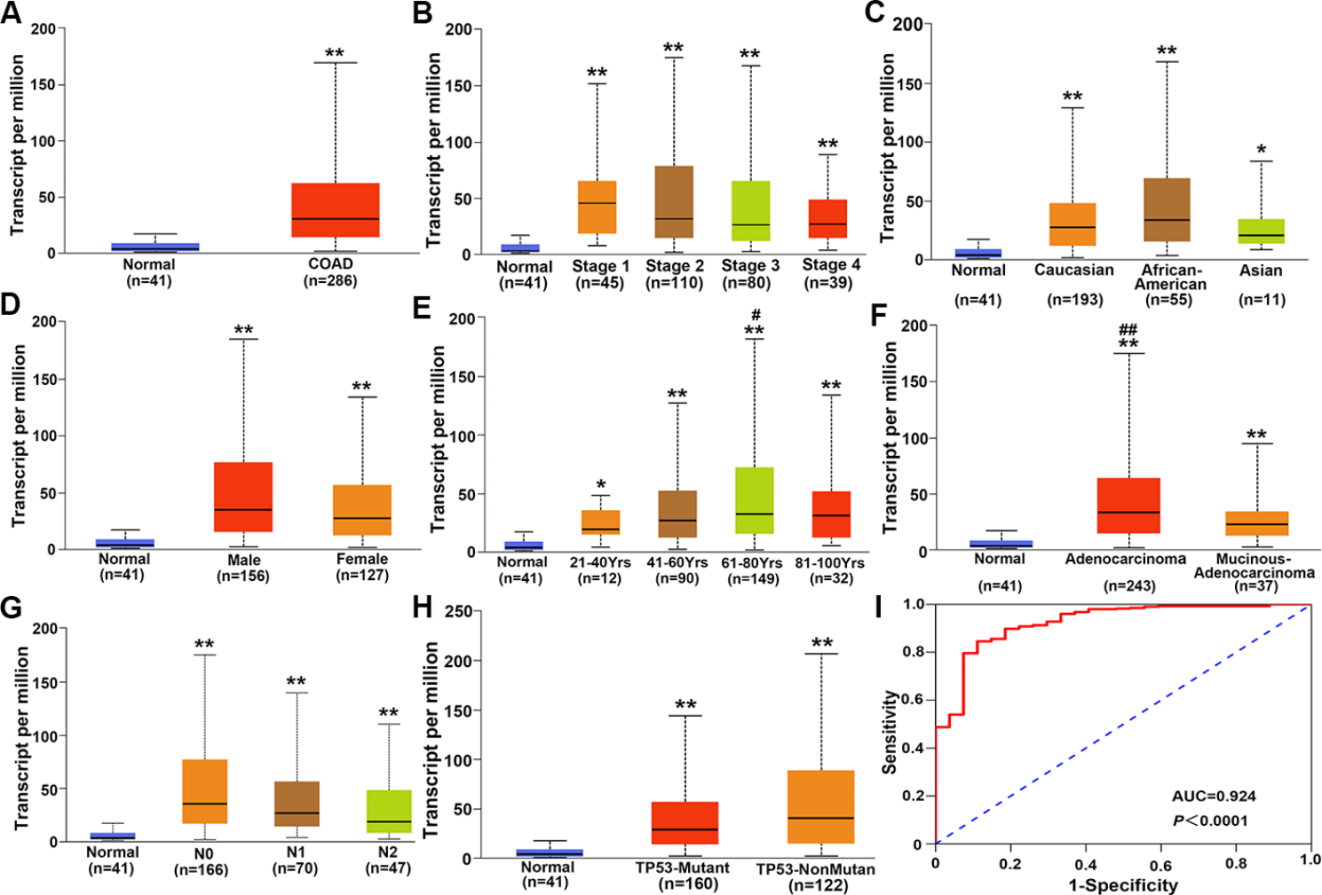
CXCL3 mRNA is highly expressed in COAD tissues, and its high expression was correlated with clinicopathological parameters. **A** Analysis of CXCL3 mRNA expression in normal and COAD tissues. (**B-H**) Analysis of CXCL3 mRNA expression in patients with different clinicopathological parameters including clinical stage, race, gender, age, histological subtype, nodal mestastasis status, and TP53 mutation status. I The diagnostic value of CXCL3 in COAD determined by ROC curve. ^*^P < 0.05 vs. Normal, ^**^P < 0.01 vs. Normal, ^#^*P* < 0.05 vs. 21–40 Yrs and 41–60 Yrs, ^##^*P* < 0.01 vs. Mucinous-adenocarcinoma

The analysis of results from TCGA-COAD data showed that the expression level of CXCL3 mRNA in COAD tissues is surprisingly higher than that in normal colon tissue (Fig. 1A). In addition, the high-level expression of CXCL3 mRNA was also related to the patient’s clinical stage (Fig. 1B), race (Fig. 1C), gender (Fig. 1D), age (Fig. 1E), histological subtype (Fig. 1F), nodal mestastasis (Fig. 1G), and TP53 mutation status (Fig. 1H). Auc (= 0.924, *P* < 0.0001) was calculated by ROC curve, indicating that the expression level of CXCL3 mRNA has very high accuracy in the clinical diagnosis of COAD (Fig. 1I).

### CXCL3 protein is highly expressed in COAD tissues

**Fig. 2:**
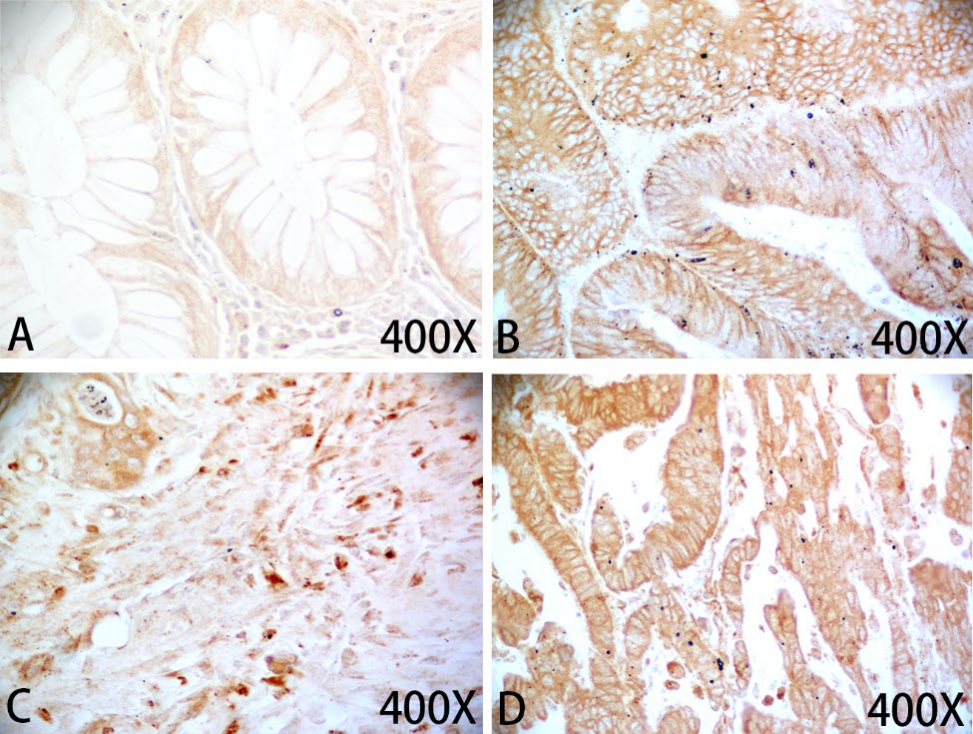
CXCL3 protein is strongly expressed in COAD tissues. **A** Weak immunohistochemical staining of CXCL3 protein in normal colon tissue. (**B-D)** Strong immunohistochemical staining of CXCL3 protein in COAD tissues with stage I (B), stage II (C), and stage III (D)

A commercial human colon tissue microarray comprising 54 normal colon and 54 COAD tissues was utilized for immunohistochemistry assay. Of the cancer tissues in the microarray, 11 with stage I, 27 with stage II, 15 with stage III, and 1 with stage IV. In addition, 9 had grade 1, 34 had grade 2 and 11 had grade tumors; 3 had T1, 10 had T2, 31 had T3 and 10 had T4 tumors. The findings revealed that high-level expression of CXCL3 protein was detected in 22.22% (12/54) of the COAD tissues, but in 7.41% (4/54) of the normal colon tissues (Fig. 2).

### CXCL3 expression is associated with tumor-associated chemokines, genes, signaling pathways and immunocyte recruitment in COAD

**Fig. 3:**
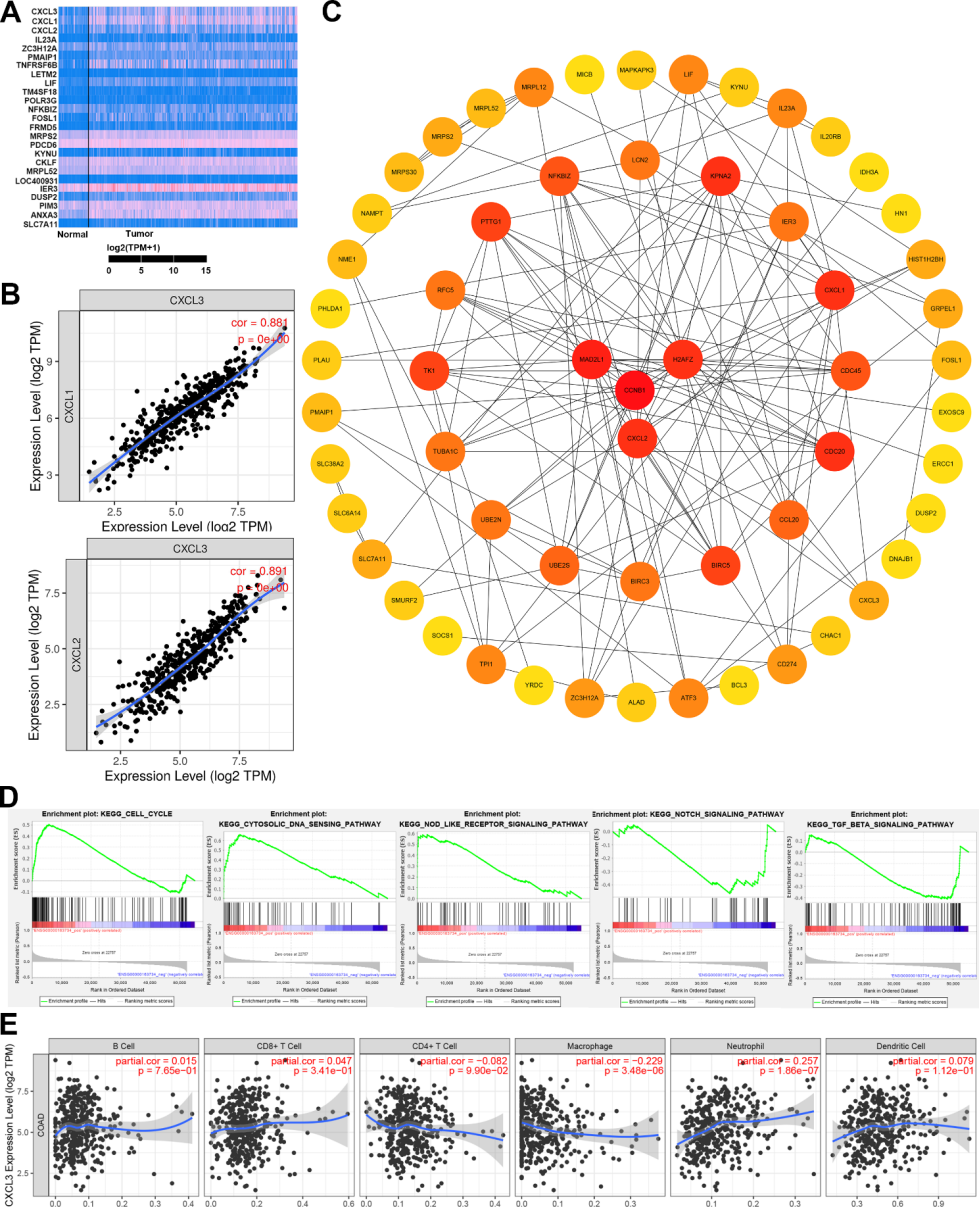
CXCL3 expression is related to tumor-associated chemokines, genes, signal pathways and immunocyte recruitment in COAD. **A** Heatmap of the top 25 CXCL3-related genes. **B** Correlation analyses between CXCL3 and the top two genes, CXCL1 and CXCL2, in the heatmap. **C** Protein interaction network (PPI) analysis of genes that closely related to CXCL3. **D** GSEA analyses of pathways that significantly affected by CXCL3. **E** Correlation analyses between CXCL3 and immunocyte recruitment

Gene expression associated assay showed that a total of 94 genes have strong positive association with CXCL3, in which the top 25 genes were displayed in the form of heatmap (Fig. 3A). To further verify above results, we analyzed the correlation between CXCL3 and the top two genes in the heatmap by using TIMER database, the findings also showed CXCL1 and CXCL2 were extremely correlated with CXCL3 expression in COAD (Fig. 3B). PPI analysis presented that CCNB1, MAD2L1, H2AFZ and CXCL2, as proteins closely related to CXCL3, may play a pivotal role for CXCL3 to exert its carcinogenic potential in COAD (Fig. 3C). Furthermore, another gene mentioned above, CXCL1, are also implicated in the CXCL3-related PPI network (Fig. 3C). In order to better understand the potential mechanism of CXCL3 in COAD, we performed GSEA analysis in gene ontology. The results revealed that CXCL3 was notably enriched in the cell cycle, and mainly involved in DNA sensing, NOD-like receptors, NOTCH and TGF-β signal pathways (Fig. 3D). In addition, further analysis of immunocyte recruitment demonstrated that CXCL3 expression was negatively correlated macrophage recruitment, but positively correlated with neutrophil recruitment (Fig. 3E).

### Exogenous CXCL3 stimulates HT-29 and SW480 cell proliferation and migration abilities

**Fig. 4:**
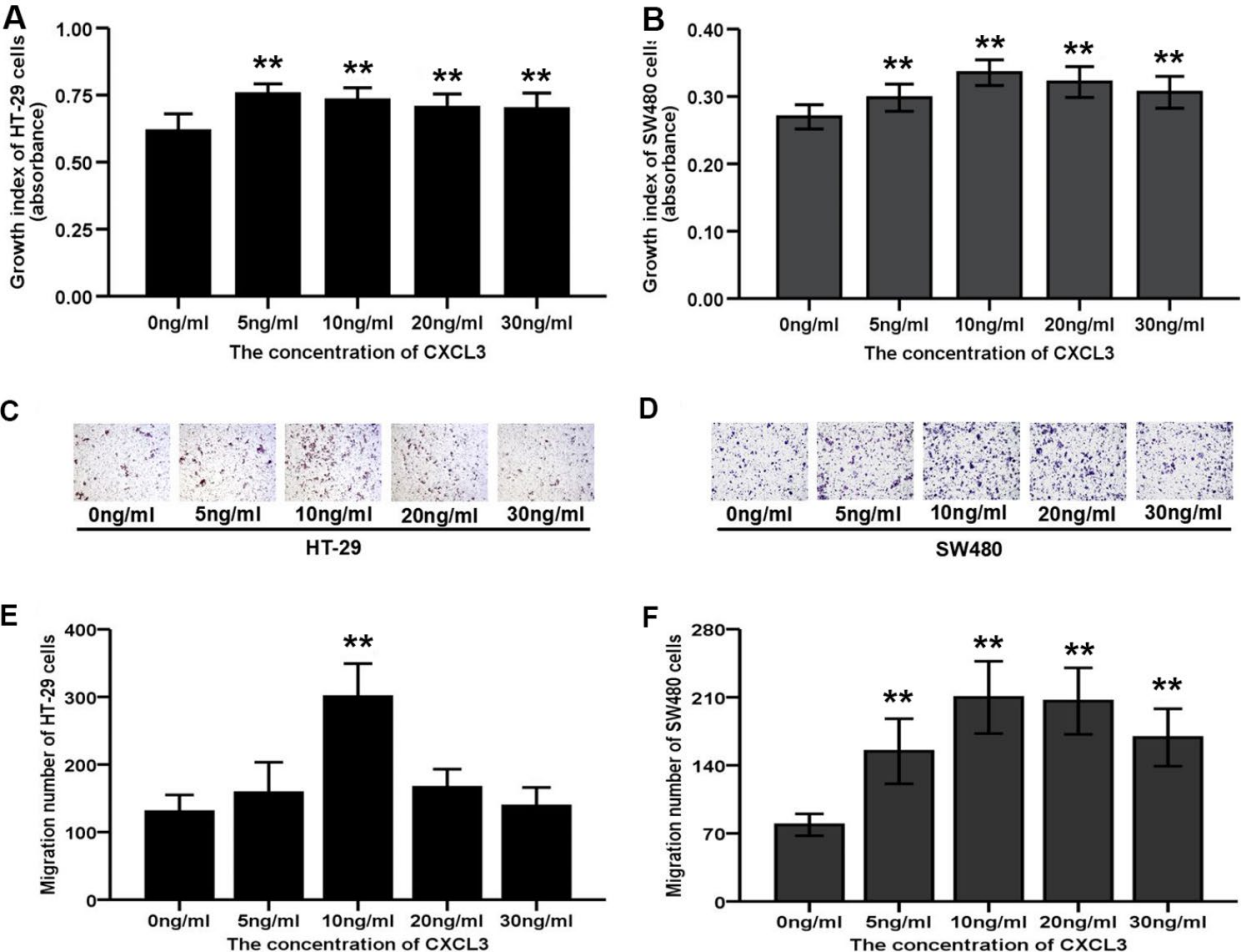
Exogenous administration of CXCL3 contributes to the malignant behaviors of COAD cells. (**A-B**) CCK-8 analysis of colon cancer cells treated with different concentrations of exogenous CXCL3. (**C-D**) Representative images of transwell assay for cells treated with different concentrations of exogenous CXCL3. (**E-F**) Transwell analysis of colon cancer cells treated with different concentrations of exogenous CXCL3. **P < 0.01 vs. 0 ng/ml

CCK-8 assays showed a significant increase in cell proliferation for HT-29 and SW480 cells following treatment with 5, 10, 20 and 30 ng/ml of exogenous CXCL3 compared with untreated cells (Fig. 4A and B). Similarly, the migration ability of HT-29 cells following treatment with exogenous CXCL3 at concentration of 10 ng/ml, as well as SW480 cells at concentration of 5, 10, 20 or 30 ng/ml was obviously enhanced (Fig. 4C-F).

### CXCL3 overexpression elevates HT-29 and SW480 cell proliferation, migration and colony formation abilities

**Fig. 5:**
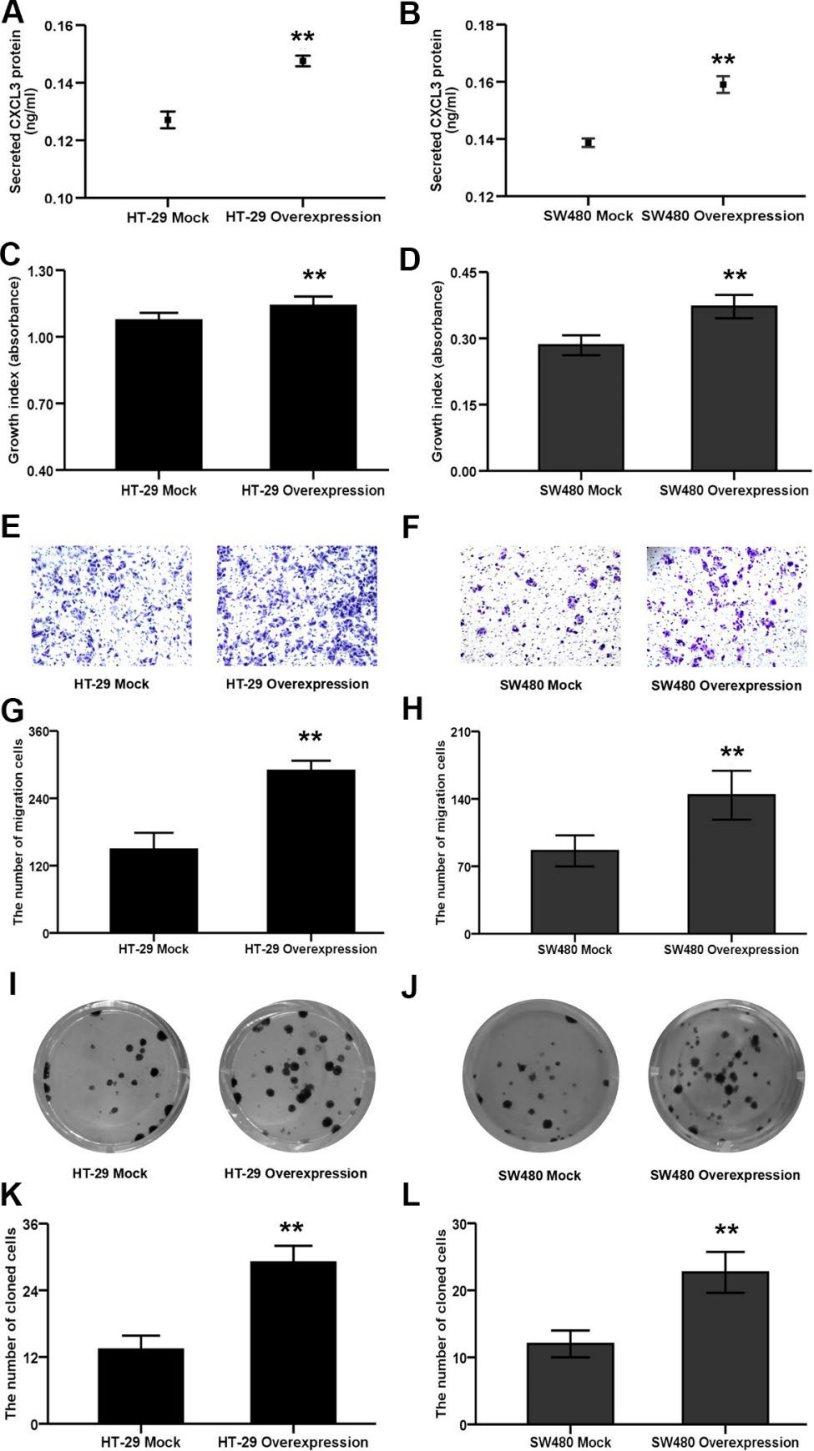
CXCL3 overexpression facilitates the malignant behaviors of COAD cells. (**A-B**) ELISA analysis of CXCL3 expression levels in the medium supernatant from CXCL3-overexpressing COAD cells and their mock control cells. (**C-D**) The analysis of cell proliferation by CCK8. (**E-F**) Representative images of transwell assay for cell migration. (**G-H**) The quantified and statistical analysis of cell migration by transwell assay. (**I-J**) Representative images of colony formation assay for cell cloning ability. (**K-L**) The quantified and statistical analysis of cell cloning ability by colony formation assay. **P < 0.01

To further assess the endogenous mechanisms through which CXCL3 affects the malignant phenotypes of COAD cells, HT-29 and SW480 cells overexpressing CXCL3 were established by gene transfection, and CXCL3-transfected cells (overexpression) and their mock control cells (mock) were identified by ELISA (Fig. 5A and B). CCK-8, Transwell and cell colony-forming assays exhibited that the proliferation (Fig. 5C and D), migration (Fig. 5E-H) and cloning formation abilities (Fig. 5I-L) of CXCL3-overexpressiing HT-29 and SW480 cells were obviously enhanced relative to their respective mock cells.

### CXCL3 deficiency inhibites HT-29 and SW480 cell proliferation, migration and colony formation abilities

**Fig. 6:**
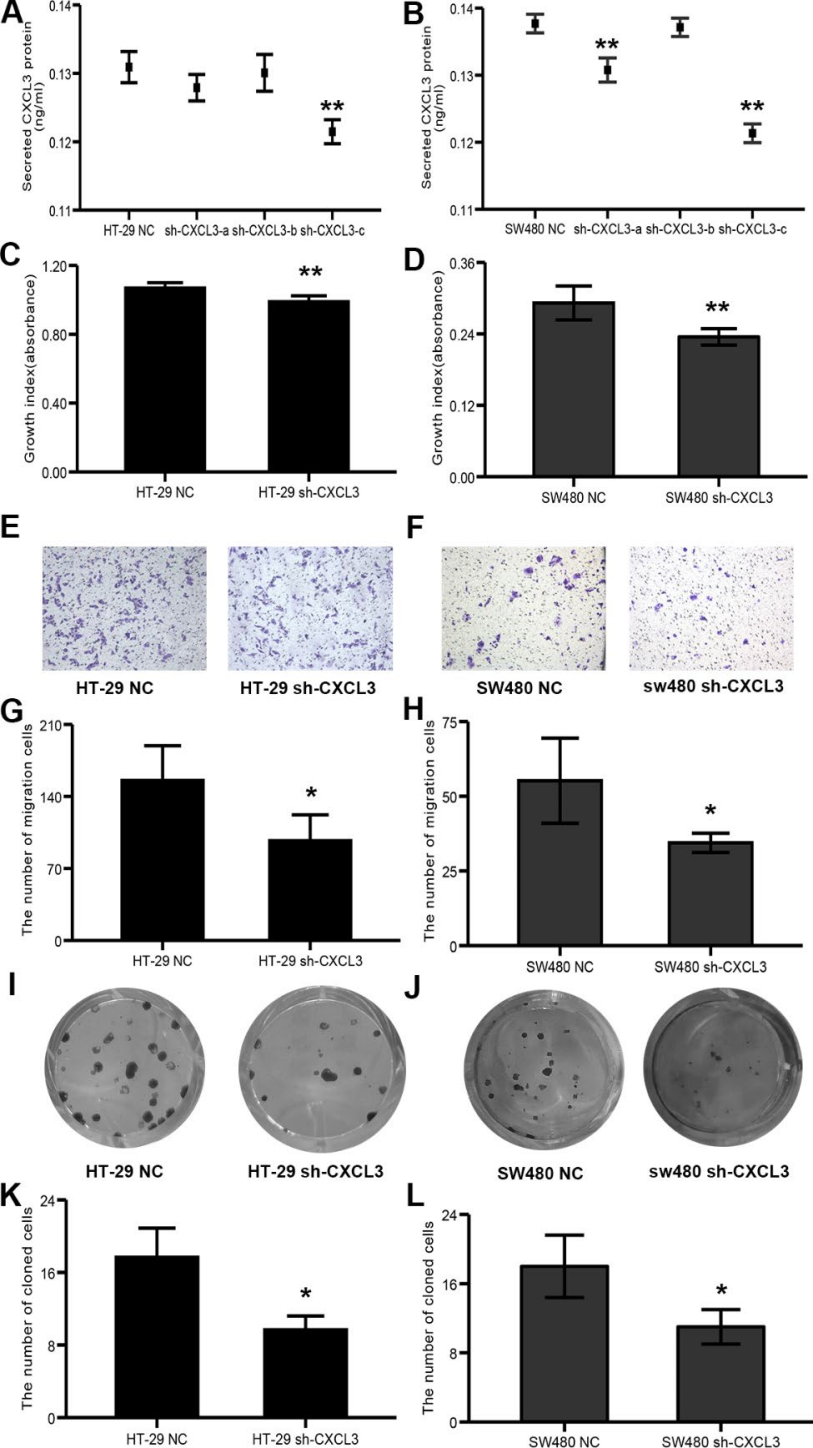
CXCL3 deficiency inhibits the malignant behaviors of COAD cells. (**A-B**) ELISA analysis of CXCL3 expression levels in the medium supernatant from CXCL3- deficiency COAD cells and their mock control cells. (**C-D**) The analysis of cell proliferation by CCK8. (**E-F**) Representative images of transwell assay for cell migration. (**G-H**) The quantified and statistical analysis of cell migration by transwell assay. (**I-J**) Representative images of colony formation assay for cell cloning ability. (**K-L**) The quantified and statistical analysis of cell cloning ability by colony formation assay. **P < 0.01, *P < 0.05

Similarly, CXCL3-deficiency cells were established by transfection with interference sequence targeting CXCL3 (Fig. 6A and B), and down-regulation of CXCL3 significantly suppressed proliferation (Fig. 6C and D), migration (Fig. 6E-H) and cloning formation abilities (Fig. 6I-L) of HT-29 and SW480 cells.

### CXCL3 overexpression regulates the expression of ERK signaling pathway related genes in HT-29 and SW480 cells

**Fig. 7:**
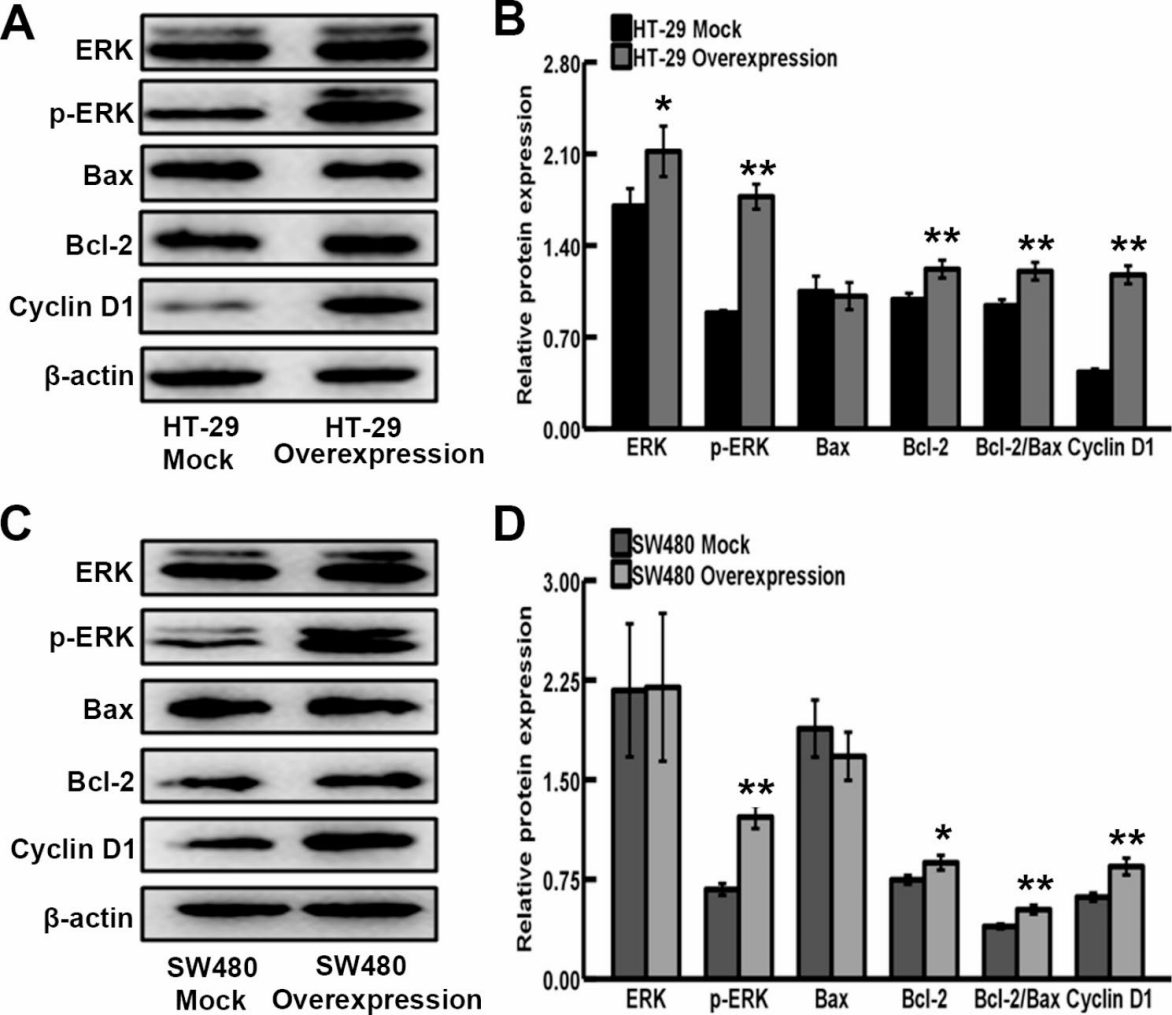
CXCL3 overexpression modulates ERK pathway-related protein expression in COAD cells. (**A, C**) Representative images of western blot assay for protein expression. (**B, D**) Analysis of protein levels (normalized to β-actin). ^*^P < 0.05 vs. Mock, ^**^P < 0.01 vs. Mock

The underlying mechanism mediating CXCL3-related malignant behaviors was evaluated by detecting gene expression at protein level using western blotting assays. As shown in Fig. 6A and B, the results presented that increased ERK, p-ERK1/2, Bcl-2, Cyclin D1 protein levels were observed in CXCL3-overexpressing HT-29 cells. Although CXCL3 overexpression in HT-29 cells did not affect Bax protein level, Bcl-2/Bax ratio was significantly increased (Fig. 7A and B). Meanwhile, our results also displayed that increased p-ERK1/2, Bcl-2, Cyclin D1 protein levels and Bcl-2/Bax ratio were observed in SW480 cells overexpressing CXCL3, but no significant difference in the protein levels of ERK and Bax was detected between CXCL3-overexpressing SW480 cells and their mock control cells (Fig. 7C and D).

### ERK inhibitor PD98059 inhibited the proliferation and migration of HT-29 and SW480 cells overexpressing CXCL3

**Fig. 8:**
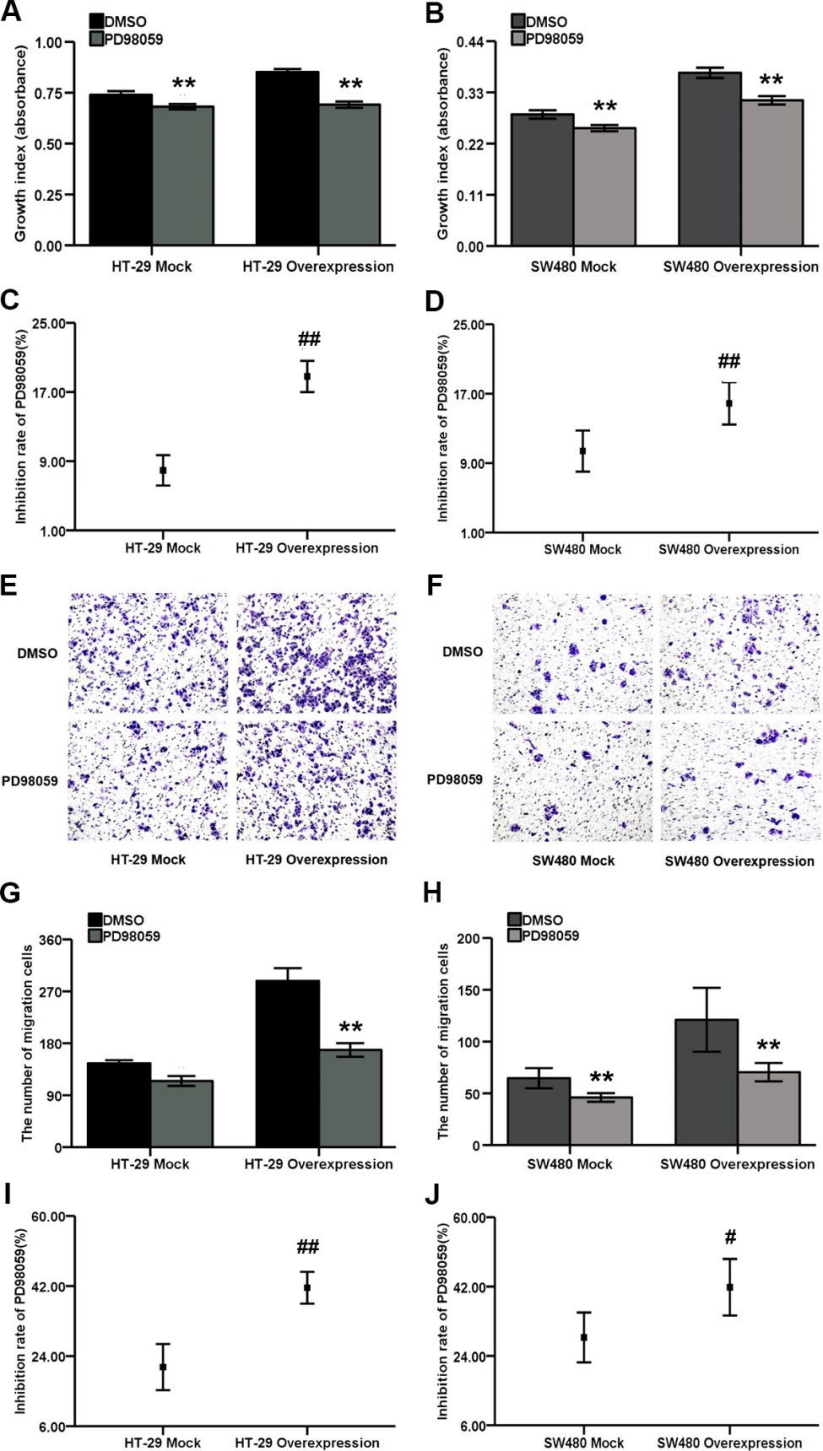
ERK inhibitor PD98059 attenuates CXCL3-induced malignant behaviors in COAD cells. (**A-B**) CCK-8 analysis for COAD cells treatment with DMSO (Control) and 100 µΜ PD98059. (**C-D**) The inhibition rate analyses of cell proliferation derived from PD98059 on CXCL3-overexpressing COAD cells and their mock control cells. (**E-F**) Representative images of transwell analysis for COAD cells treatment with DMSO (Control) and 100 µΜ PD98059. (**G-H**) transwell analysis for COAD cells treatment with DMSO (Control) and 100 µΜ PD98059. (**I-J**) The inhibition rate analyses of cell migration derived from PD98059 on CXCL3-overexpressing COAD cells and their mock control cells. ^**^P < 0.01 vs. DMSO, ^##^*P* < 0.01 vs. MOCK, ^#^*P* < 0.05 vs. MOCK.

The biological behaviors of COAD cells in response to ERK1/2 blocker PD98059 were assessed by CCK-8 and Transwell assays. The results demonstrated proliferation (Fig. 8A and B) and migration (Fig. 8E-H) abilities of HT-29 and SW480 cells, whether or not to overexpress CXCL3, were significantly reduced following treatment with 100 µΜ PD98059, suggesting an involvement of ERK1/2 pathway in the malignant process of COAD. In addition, the effects of PD98059 on the proliferation (or migration) inhibition rate of cells was determined as follows: proliferation (or migration) inhibition rate (%) = [absorbance (or the number of migratory cells) upon treatment with DMSO - absorbance (or the number of migratory cells) upon treatment with 100 µΜ PD98059] / absorbance (or the number of migratory cells) upon treatment with DMSO × 100%. The results showed that the inhibition rates exerted by PD98059 on CXCL3-overexpressing cell proliferation (Fig. 8C and D) and migration (Fig. 8I and J) were surprisingly enhanced compared with their mock control cells, indicating the contribution of CXCL3 to the pathogenesis of COAD via an ERK-dependent mechanism.

## Discussion

Chemokine CXCL3, also known as growth related gene 3 (GRO3), GROγ or Macrophage inflammatory protein-2-β (MIP-2β), is a single-chain protein which is encoded by human growth-related oncogene (GRO) located in the chromosomal region of 4q21 [17]. CXCL3 is an important mediator of inflammation response that can attract distinct populations of cells, particularly endothelial cells, epithelial cells, and fibroblasts, to damaged sites in a ligand and receptor-dependent manner [18]. Recently, it has been increasingly recognized that CXCL3 appears to enhance tumor angiogenesis and has a key role in tumor cell growth, migration, invasion, and metastasis through several mechanisms, including inducing calcium mobilization and ERK1/2 phosphorylation, inhibiting the activation of adenylate cyclase activator, and reducing the generation of cyclic adenylate [19]. Accordingly, our previous studies also revealed that CXCL3 facilities the malignant behaviors of tumor cells in cervical cancer and prostate cancer via activation of the MAPK/ERK signaling pathway [10, 11].

In this study, the results from bioinformatics and immunohistochemistry assays exhibited that CXCL3 mRNA and protein are surprisingly upregulated in COAD tissues, and its high-level expression is closely correlated with several clinicopathological parameters, including patient’s clinical stage, race, gender, age, histological subtype, nodal mestastasis and TP53 mutation status. Our results also presented that CXCL3 expression has a strong positive association with CXCL1 and CXCL2 expression, as well as neutrophil attraction, but has a negative association with macrophage attraction. Chemokines are best known for their roles in mediating the attraction of distinct populations of immune cells that are responsible for the balance between pro- and anti-tumorigenic immune responses. The important immune cells implicated in the elimination of tumor are natural killer (NK) cells, T helper 1 (Th1) cells, monocytes and CD8 + T cells, whereas several immune cell subsets including neutrophils, myeloid-derived suppressor cells (MDSC) and regulatory T cells (Treg) have the ability to facilitate tumor development [20]. CXCL1, CXCL2 and CXCL3, also known as GROα, GROβ, and GROγ, are the three extremely homologous subtypes that belong to a superfamily of chemokine growth related oncogenes. Increasing evidence suggested that CXCL1, CXCL2 and CXCL3 are the main chemokines that directly or indirectly affect the shaping of the TME through their role in mediating the recruitment of populations of immune cells under conditions of inflammatory and immunological responses [21]. Consistent with this, our findings in the aforementioned study indicated that the crosstalk among CXCL3 CXCL1 and CXCL2 may regulate the phenotype and function of immune cells by mediating their recruitment and cellular interactions in the TME of COAD. Subsequently, PPI and GSEA assays indicated that genetic and cell biological aspects including CCNB1, MAD2L1, H2AFZ, CXCL2 genes and cell cycle, as well as several signal pathways including DNA sensing, NOD-like receptors, NOTCH and TGF-β signal pathways may be involved in CXCL3-mediated tumor biology in COAD. Furthermore, we also discovered in vitro that exogenous administration or overexpression of CXCL3 remarkably enhanced malignant behaviors of HT-29 and SW480 cells, whereas down-regulation of CXCL3 exerted opposite effect on cells. Intriguingly, the migratory potential of HT-29 cells decreased after treament with 20 or 30 ng/ml CXCL3, despite significant cell proliferation compared to the untreated cells at these doses. In a previous study, we found that the proliferation and migration of prostate cancer cells show obvious dose-dependent effect with CXCL3 concentration increased [11]. However, when the dose of CXCL3 increases to a certain value, it actually inhibits malignant behaviors of these tumor cells and induces down-regulated expression of ERK, suggesting the inhibitory effect exertd by high doses of CXCL3 may be related to its blockade on the ERK signal [11]. In additon, we also speculated that this phenomenon may be related to the fact that high-level ligands reduce affinity of the receptors to them [10]. Although the current study did not detect the effect of CXCL3 on the proliferation and migration of normal colon cells, we have confirmed in previous study that CXCL3 also exerts proliferative and migration-promoting roles on non-tumor cells [11], suggesting that CXCL3 has not a specific role in tumor cells.The mechanisms underlying the tumor process are related numerous signal pathways, in which mitogen activated protein kinase (MAPK) signal pathway is essential for the growth and diffusion of human cancer cells [22]. MAPK pathway is highly complex and is composed of three subfamilies, in which MAPK/ERK (extracellular signal regulated kinase) pathway has been shown to function as an integrating node of converging signaling pathways. Phosphorylation is a critical step for the activation of ERK1/2, and this event additively trigger translocation of p-ERK1/2 into nucleus where p-ERK1/2 is necessary for the phosphorylation/activation of various nuclear transcription factors and protein kinase substrates that are directly related to the process of cell proliferation and differentiation [23]. It has been demonstrated that specific intereactions of chemokines with their receptors in human tumors is mediated by the MAPK/ERK signaling pathway. For example, Hu et al. highlighted a crucial link between CXCL12-CXCR7 interactions in skin squamous carcinoma cells, through which tumor cells obtained the ability to enhance their malignant phenotypes in an ERK-depentent manner [24]. More recently, it was found that ERK signal may be involved in the potential contribution of CXCL12/CXCR4 to survival of cancer cells and chemoresistance in glioblastoma [25]. The current study displayed that high expression of CXCL3 in COAD cells results in increased expression of ERK. Based on this conclusion, we further employed ERK blocker PD98059 to assess whether upregulation of CXCL3 expression enables tumor cells to specififically activate ERK signal pathway that facilitates the process of COAD. The results demonstrated that CXCL3-induced malignant behaviors in HT-29 and SW480 cells were predominantly restrained following blocking of ERK, suggesting an involvement of ERK signal pathway in in CXCL3-associated tumor biology in COAD.

In addition, our results showed that CXCL3 overexpression also exerts its role to regulate Bcl-2, Bcl-2/Bax and Cyclin D1 expression that are responsible for apoptosis and cell cycle, the processes upon which tumor cells depend for proliferation, survival, and metastasis. Apoptosis is an energy-dependent cell suicide event that is strictly regulated by several apoptosis-related gene families, in which Bcl-2 family plays a key role. Bcl-2 family includes both pro- and anti-apoptotic genes, and the relative ratio of pro-/anti-apoptotic genes, especially Bcl-2/Bax, is considered to be a major determinant for the balance between cell survival and death [26]. Numerous studies have focused on the understanding of the ERK signal controlling the function and expression of Bcl-2 gene and its family gene Bax in human tumors [27, 28]. For instance, a search for chemokine/chemokine receptor system in lung cancer showed that the interaction of CC-chemokine ligand 21 (CCL21) with its receptor CC-chemokine receptor 7 (CCR7) may help protect against tumor cell apoptosis through p-ERK-mediated increase of the Bcl-2/Bax ratio [29]. Cyclin D1 is an important G1/S phase modulator for cell cycle control, its overexpression and dysregulation in cancer are the frequent events that may be sufficient for many malignant tumor development [30]. Cyclin D1-driven tumorigenesis requires the activation of various signal pathways including ERK pathway [31, 32]. For example, Fan et al. revealed a possible cooperation of Cyclin D1 with ERK, they found that overexpresion of NEK2 resultes in the upregulation of Cyclin D1 in a MAPK/ERK-dependtent manner, upon with tumor cells tumor the ability to regulate the process of cell mitosis, and eventually leads to increased proliferative response [33].

## Conclusion

In summary, our experiment showed that chemokine CXCL3 is highly expressed in COAD, and its high-level expression is closely associated with several tumor-associated clinicopathological parameters, chemokines, genes, signaling pathways and immunocyte recruitment. In vitro, CXCL3 plays a crucial role in promoting the carcinogenic potential of COAD cells through endogenous and exogenous pathways. Mechanically, ERK、Bcl-2 and Cyclin D1, the MAPK/ERK pathway related genes that are most notably related to the apoptosis and cell cycle, are intimately involved in CXCL3-mediated malignant behaviors. Our findings indicated that activation of MAPK/ERK signal pathway controlled by upregulation of CXCL3 might be one of the key determinants in tumor biology of COAD.

### Electronic supplementary material

Below is the link to the electronic supplementary material.

Supplementary Material 1

## Data Availability

The data from bioinformatics analysis were originated from TCGA (https://portal.gdc.cancer.gov/), UALCAN (http://ualcan.path.uab.edu/), String (https://cn.string-db.org/) and TIMER (https://cistrome.shinyapps.io/timer/) open-access databases.
