# Supplementary material for: Elevated expression of CXCL3 in colon cancer promotes malignant behaviors of tumor cells in an ERK-dependent manner
Source: BMC Cancer. 2023 Nov 29;23:1162. doi: 10.1186/s12885-023-11655-y (PMC10685652; doi:10.1186/s12885-023-11655-y)

**Fig 7A**

1-1 fig7A-1 HT-29 ERK 0.3s


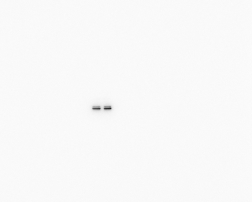


1-2 fig7A-1 HT-29 ERK 0.4s


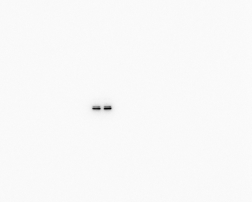


1-3 fig7A-1 HT-29 ERK 0.5s


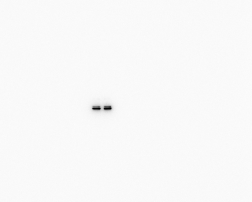


1-4 fig7A-1 HT-29 ERK pvdf


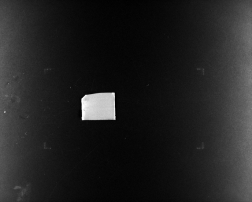


2-1 fig7A-2 HT-29 p-ERK 10s


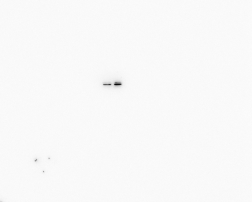


2-2 fig7A-2 HT-29 p-ERK 15s


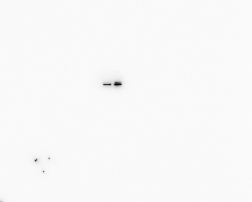


2-3 fig7A-2 HT-29 p-ERK 20s


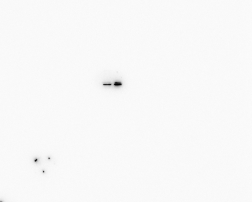


2-4 fig7A-2 HT-29 p-ERK pvdf


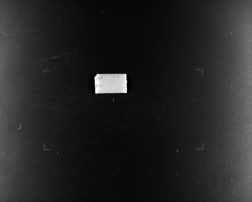


3-1 fig7A-3 HT-29 Bax 20s


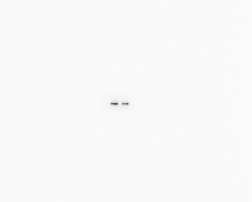


3-2 fig7A-3 HT-29 Bax 30s


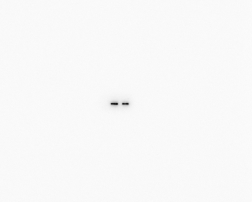


3-3 fig7A-3 HT-29 Bax 40s


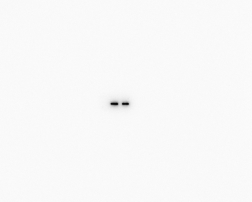


3-4 fig7A-3 HT-29 Bax pvdf


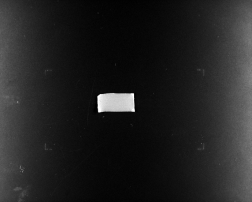


4-1 fig7A-4 HT-29 Bcl-2 10s


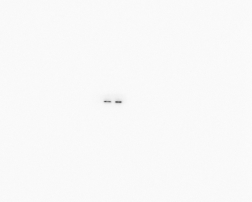


4-2 fig7A-4 HT-29 Bcl-2 15s


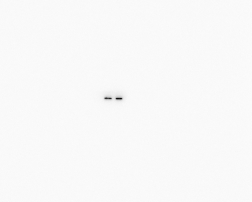


4-3 fig7A-4 HT-29 Bcl-2 20s


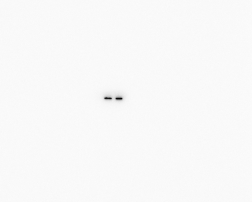


4-4 fig7A-4 HT-29 Bcl-2 pvdf


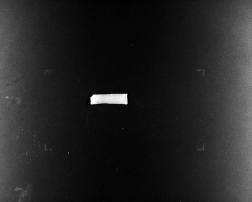


5-1 fig7A-5 HT-29 Cyclin D1 60s


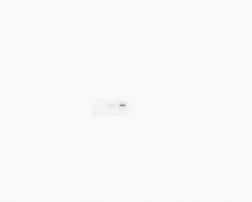


5-2 fig7A-5 HT-29 Cyclin D1 100s


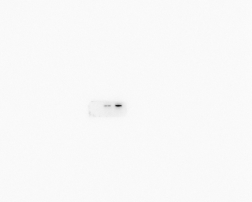


5-3 fig7A-5 HT-29 Cyclin D1 120s


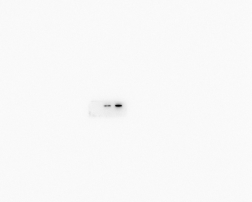


5-4 fig7A-5 HT-29 Cyclin D1 pvdf


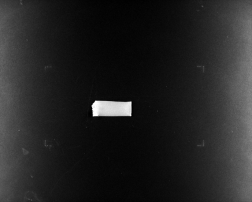


6-1 fig7A-6 HT-29 actin 4s


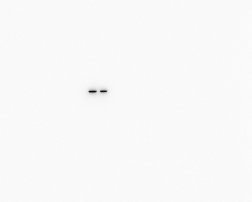


6-2 fig7A-6 HT-29 actin 5s


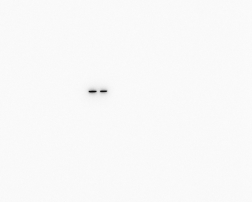


6-3 fig7A-6 HT-29 actin 6s


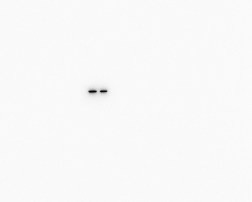


6-4 fig7A-6 HT-29 actin pvdf


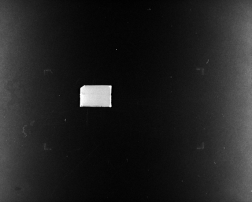


**Fig 7C**

- 1. fig7C-1 SW480 ERK 10s


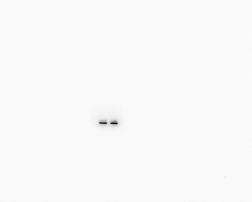


- 1. fig7C-1 SW480 ERK 12s


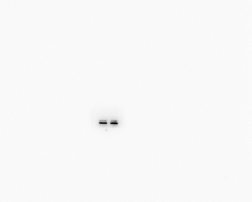


- 1. fig7C-1 SW480 ERK 15s


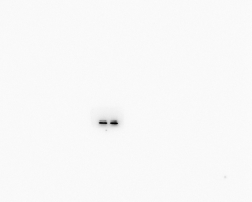


- 1. fig7C-1 SW480 ERK 20s


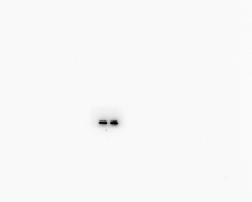


- 1. fig7C-1 SW480 ERK pvdf


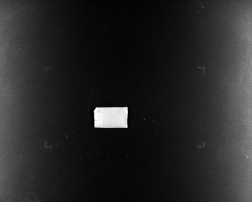


2-1 fig7C-2 SW480 p-ERK 0.5s


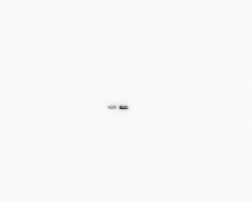


2-2 fig7C-2 SW480 p-ERK 1s


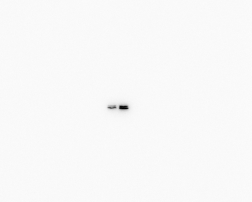


2-3 fig7C-2 SW480 p-ERK 2s


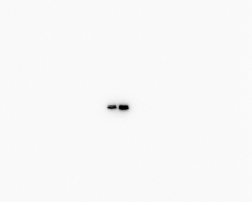


2-4 fig7C-2 SW480 p-ERK pvdf


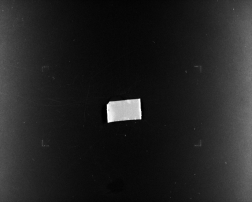


3-1 fig7C-3 SW480 Bax 3s


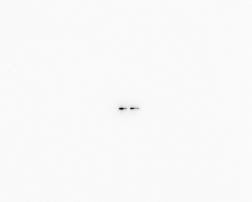


3-2 fig7C-3 SW480 Bax 5s


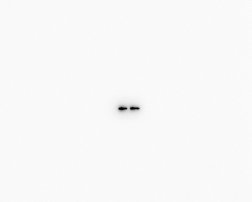


3-3 fig7C-3 SW480 Bax 10s


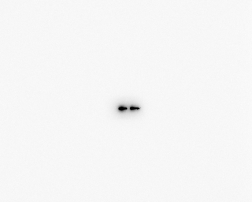


3-4 fig7C-3 SW480 Bax pvdf


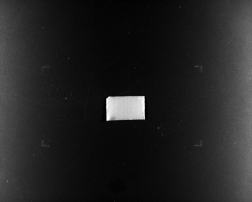


4-1 fig7C-4 SW480 Bcl-2 60s


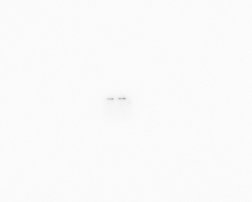


4-2 fig7C-4 SW480 Bcl-2 180s


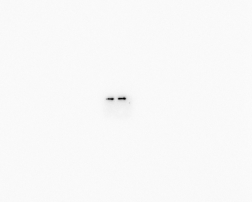


4-3 fig7C-4 SW480 Bcl-2 pvdf


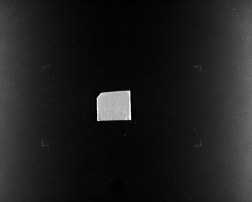


5-1 fig7C-5 SW480 Cyclin D1 8s


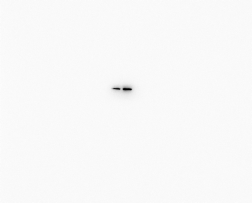


5-2 fig7C-5 SW480 Cyclin D1 10s


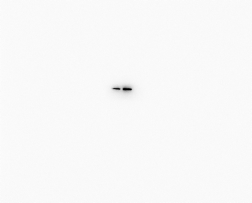


5-3 fig7C-5 SW480 Cyclin D1 pvdf


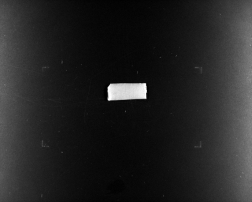


6-1 fig7C-6 SW480 actin 3s


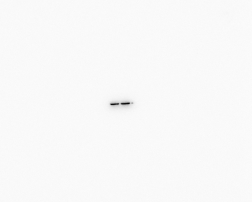


6-2 fig7C-6 SW480 actin 4s


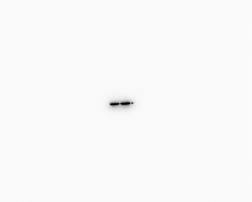


6-3 fig7C-6 SW480 actin pvdf


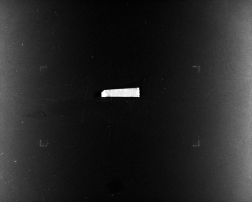

Supplement: Supplementary file 1 — Supplementary Material 1 [file 12885_2023_11655_MOESM1_ESM.docx]
